# Uncommon Cause of Right Upper Quadrant Pain Treated by Laparoscopic Surgery: A Case Report

**DOI:** 10.3389/fsurg.2022.891366

**Published:** 2022-05-09

**Authors:** Qingbo Feng, Jinpeng Du, Wenwei Liao, Jinli Zheng, Yong Zeng, Jiaxin Li

**Affiliations:** Department of Liver Surgery and Liver Transplantation Centre, West China Hospital, Sichuan University, Chengdu, Sichuan, China

**Keywords:** fish bone, gastric perforation, laparoscopic surgery, right upper quadrant pain, case report

## Abstract

**Introduction:**

Right upper quadrant pain is a very common symptom of cholecystitis. Right upper quadrant pain caused by fish bone perforation of the stomach wall is rare.

**Case Presentation:**

We report a 42-year-old woman who was admitted to our hospital with “1-month history of dull progressive right upper quadrant pain radiating to the back.” Computed tomography of the abdomen revealed a linear, high-density body between the stomach wall and the liver. On history, the patient stated she had eaten a bony fish a month prior but did not note any significant pain at the time. Laparoscopy revealed a fish bone 2 cm in length half on the surface of the caudate lobe of the liver, and no perforation of the gastrointestinal tract was found. The postoperative course was uncomplicated, and the patient was discharged home on day 3 after surgery.

**Conclusion:**

The case of right upper quadrant pain caused by the fish bone is very rare. Radiological examinations play a significant role in the diagnosis of fish bone ingestion. Laparoscopic surgery is technically feasible and safe for the treatment of patients with fish bone ingestion.

## Introduction

Right upper quadrant pain is a very common symptom of cholecystitis and is caused by fish bone perforation of the stomach wall, which is rare. Accidental ingestion of foreign bodies is very common, and the vast majority of ingested foreign bodies pass without obstruction in the gastrointestinal tract uneventfully within 1 week (1). However, some sharp foreign bodies will lead to complications. The main complications of gastrointestinal foreign bodies include bleeding, obstruction, and perforation. Among them, gastrointestinal perforation caused by gastrointestinal foreign bodies accounts for only about 1%. In general, fish bones are the most common foreign bodies, and the pylorus, the duodenojejunal flexure, the terminal ileum, and the cecum are the common sites of perforation (2). Fish bone perforation often causes liver abscess and presents with fever and vague epigastric discomfort (3–8). Perforation of the fish bone through the stomach into the liver and not causing fever or liver abscess are extremely uncommon. This report presents a rare case of the right upper quadrant pain caused by fish bone and successfully treated by laparoscopic management.

## Case Presentation

**Figure 1:**
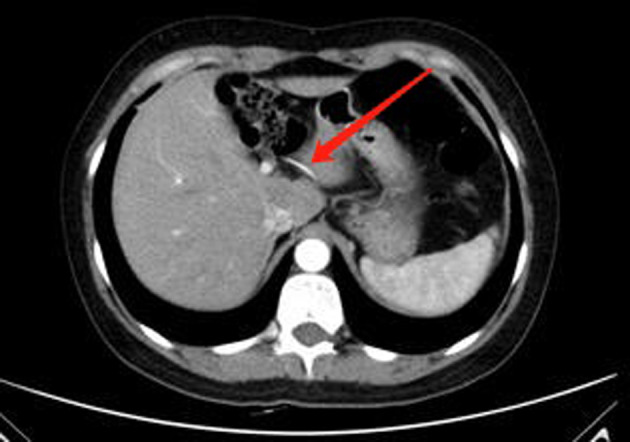
Computed tomography scan showing a hyperdense linear foreign body (red arrow) embedded in the liver.

**Figure 2:**
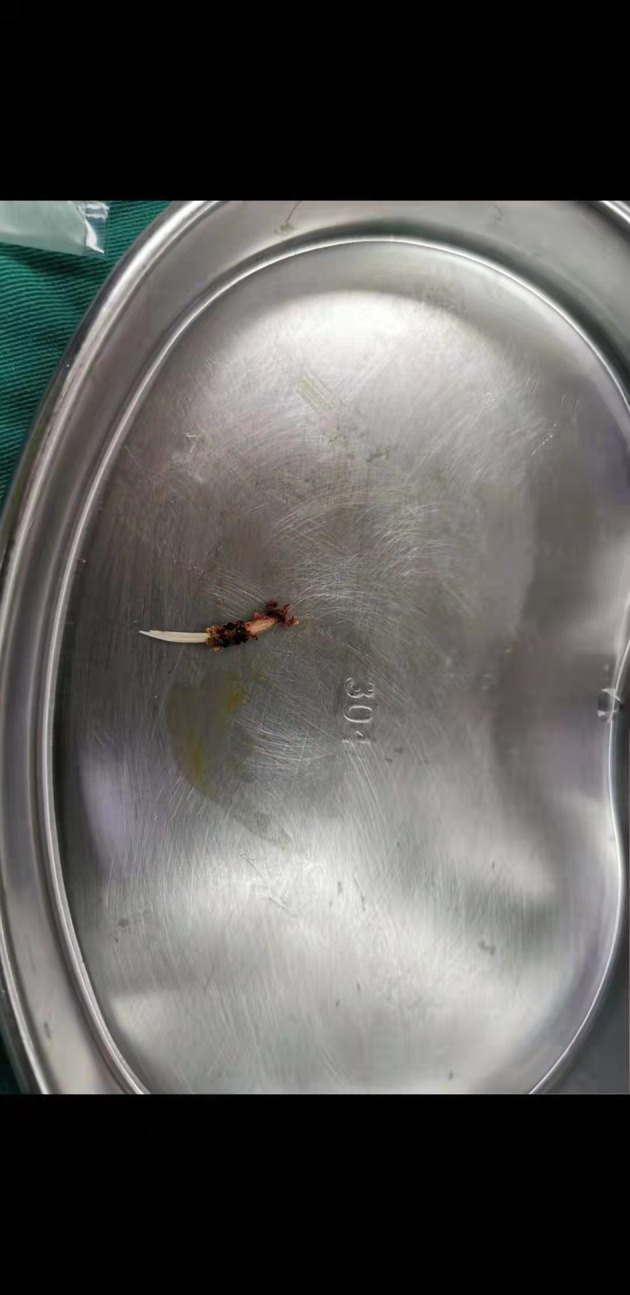
Fish bone of length 2 cm and diameter 0.2 cm extracted from the liver.

A 42-year-old female was admitted to the hospital with “1-month history of dull progressive right upper quadrant pain radiating to the back.” Upon physical examination, her abdomen was flat and soft with mild tenderness in the right upper abdomen. During imaging examinationby abdominal CT examination, a linear, high-density body in the liver caudate lobe was found. There was no evidence of abscess or pneumoperitoneum (**Figure 1**). No perforation was found by a gastroscope. In laboratory examination, the white cell count was 4.29 × 10^9^/L and deranged liver function tests showed total bilirubin of 14.3 µmol/L, alanine transaminase of 51 IU/L, and gamma-glutamyl transferase of 47 IU/L. Previous history showed that she was fit and well with no significant medical history. The patient recalled that she had eaten a bony fish a month prior but did not note any significant pain at the time. Diagnostic laparoscopy was performed under general anesthesia. During the operation, we found a fish bone inside the liver, the tail of fish bone was exposed outside the liver, and granuloma was formed around it, and the fish bone was removed laparoscopically (**Figure 2**). We did not find any signs of gastric perforation. Anti-infection was given symptomatic treatment after the operation. The patient was discharged home on day 3 after surgery. The patient recovered uneventfully without signs of recurrence 3 months after the surgery, and the liver function was normal (total bilirubin 11.2 µmol/L, alanine transaminase 11 IU/L, and gamma-glutamyl transferase 16 IU/L) in the follow-up.

## Discussion

Approximately 71% of foreign-body perforations occur in the intra-abdomin, and the remaining 29% are in the anus or distal rectum (9). Theoretically, perforation can occur anywhere along the gastrointestinal tract; however, it usually occurs at areas of angulation or narrowing, such as the pylorus and ileocecal junction. Perforation of the digestive tract caused by ingested fish bone is relatively uncommon. Enterohepatic migration of an ingested fish bone to the liver is extremely rare as the liver is not in continuity with the gastroenteric lumen. Most patients with fish bone embedded in the liver present with liver abscesses before definitive diagnosis. The most common symptoms in these patients are fever and epigastric pain. CT examination has a certain value in the diagnosis of foreign bodies. In the literature, the majority of the liver abscess caused by fish bone is detected by CECT/MRI abdomen. Accurate preoperative diagnoses of fish bone ingestion based on CT are beneficial to the operation of laparoscopic resection. Fortunately, the fish bone was removed by laparoscopic management successfully in our case.

Several studies have reported the discovery of a fish bone through the stomach into the liver, and the left liver lobe was the most involved region. The caudate lobe of the liver is rarely involved; only four cases have been reported (3), and only our case did not develop into a liver abscess.

In conclusion, we presented a rare case of fish bone perforation of the gastrointestinal tract with penetration of the liver parenchyma, which caused right upper quadrant pain. Our case demonstrated that early diagnosis and retrieval of fish bone is essential for avoiding morbidity and mortality. Laparoscopy is technically feasible and safe for the treatment of patients with fish bone ingestion.

## Data Availability

The original contributions presented in the study are included in the article/supplementary material; further inquiries can be directed to the corresponding author/s.
